# Exploring internal structures and properties of terpolymer fibers via real-space characterizations

**DOI:** 10.3762/bjnano.14.83

**Published:** 2023-10-05

**Authors:** Michael R Roenbeck, Kenneth E Strawhecker

**Affiliations:** 1 Department of Marine Engineering, U.S. Merchant Marine Academy, Kings Point, New York 11024, United States; 2 DEVCOM, Army Research Laboratory, Aberdeen Proving Ground, MD, 21005, United States

**Keywords:** atomic force microscopy, correlative characterization, high-performance fibers, structure–property relationships, Technora^®^

## Abstract

While significant research has investigated the processing and properties of high-performance terpolymer fibers, much remains to be understood about the internal nano- and microstructures of these fibers, and how these morphologies relate to fiber properties. Here we use a focused ion beam notch technique and multifrequency atomic force microscope mapping to characterize the internal structure and local mechanical properties within Technora^®^ fibers. We find a highly fibrillated structure that appears to connect with both the fiber’s molecular chemistry and full-fiber mechanical properties. In addition, through detailed comparisons with Kevlar^®^ K29 fibers, we find remarkable differences between the internal structures of the two fibers, and posit connections between our measurements and multifunctional performance studies from the literature.

## Introduction

**Figure 1:**
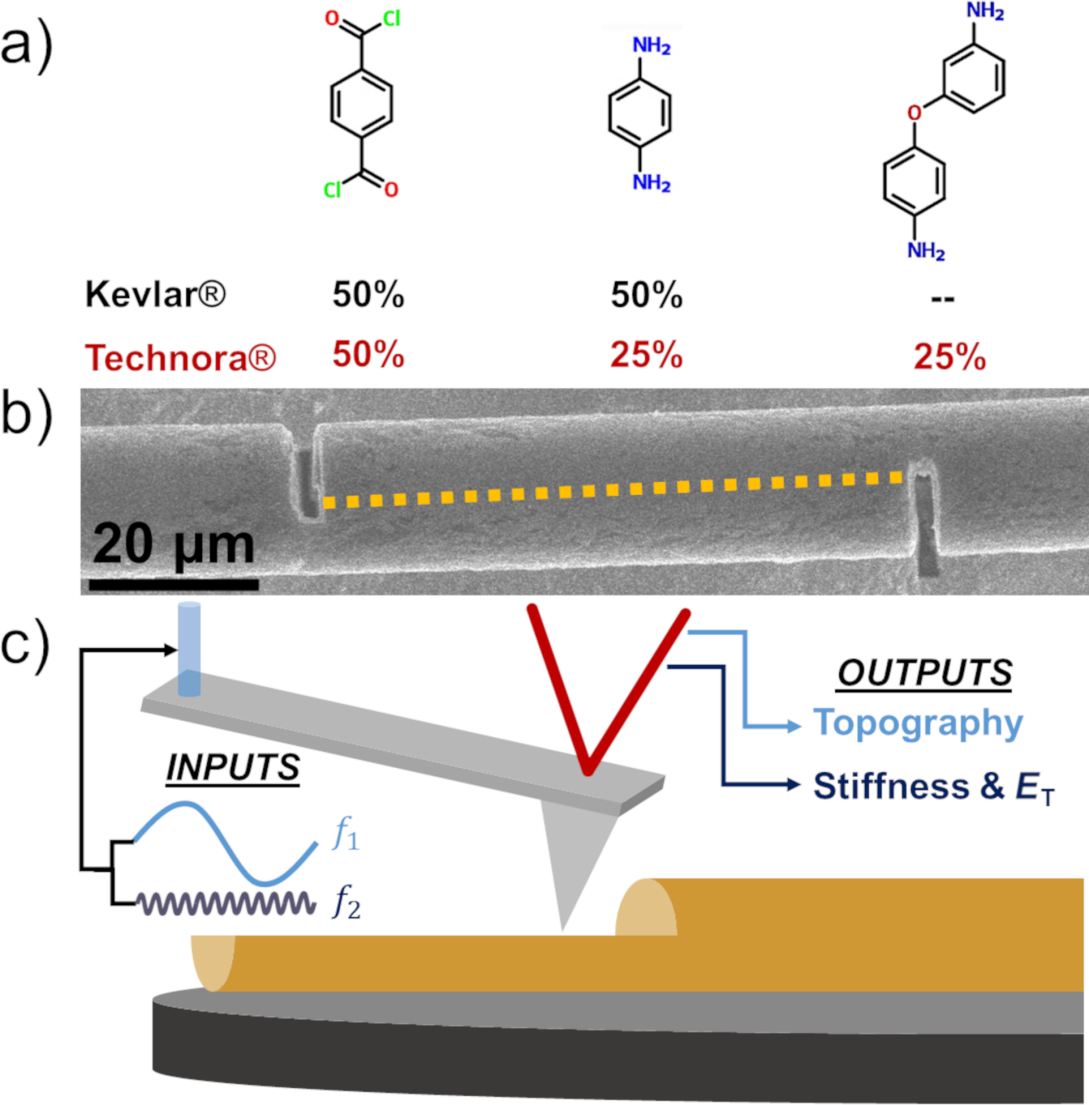
Materials and methods overview. (a) Three primary monomers utilized in Technora^®^ fibers: terephthaloyl chloride (TPA), *p*-phenylenediamine (PPD), and 3,4′-diaminodiphenyl ether (DPE), from left to right in the ratios shown [1–4]. Corresponding percentages also shown for Kevlar^®^. (b) Micrograph of a FIB-notched Technora^®^ fiber. Dashed line highlights a shear plane between the two notches. The fiber readily opens along this plane, exposing internal fiber surfaces for characterization. (c) Multifrequency AFM schematic. A cantilever is driven by a blue laser that simultaneously excites the cantilever at its first and second resonance frequencies (*f*_1_ and *f*_2_). The response of the cantilever as it scans across the exposed internal fiber surface provides information about the local fiber internal morphology and mechanical response (stiffness and transverse elastic modulus (*E*_T_)).

High-performance polymer fibers have enabled groundbreaking advancements in numerous applications, from personal armor to tires to sports equipment, that aim to maximize mechanical performance while minimizing weight. The successes achieved with forerunners in the field, such as Kevlar^®^ and ultrahigh molecular weight polyethylene (UHMWPE), have driven tremendous interest in new fiber chemistries and processing techniques. The development of Technora^®^ in 1987 marked a new approach to polymer fiber design, with an emphasis on aligning extended molecular chains rather than optimizing crystallites. This strategy aimed to explore new chemistries that could enhance multifunctional aspects of fibers without adversely affecting fiber mechanical properties. Indeed, in developing Technora^®^, Teijin Ltd. had four principal aims: to manufacture a (i) cost-effective fiber with (ii) similar mechanical properties to Kevlar^®^ that was (iii) heat-resistant and (iv) soluble in organic solvents [1]. A highly oriented fiber structure exhibiting these characteristics was achieved through a terpolymer chemical formulation, consisting of terephthaloyl chloride (TPA), *p*-phenylenediamine (PPD), and 3,4′-diaminodiphenyl ether (DPE) (Figure 1a). Compared to Kevlar^®^, which includes 50% concentrations of the first two monomers alone (forming *p*-phenylene terephthalamide (PPTA)), the molecular structure of Technora^®^ substitutes DPE for half of the PPD monomers [1–5]. Of course, this distinction in fundamental chemistry has significant implications for the structures and properties of the resulting fibers.

To date, structure–property characterizations of Technora^®^ in the literature have primarily focused on (i) X-ray diffraction (XRD), (ii) nuclear magnetic resonance (NMR) spectroscopy, and (iii) fiber tensile properties. Interestingly, although Technora^®^ is noncrystalline, it can be effectively modeled through XRD as a paracrystalline material such as Kevlar^®^. Within this framework, the Technora^®^ paracrystalline distortion parameter, a measure of crystalline “sinuosity”, was lower (i.e., indicated better alignment) than in all Kevlar^®^ classes explored in a study by Wu and Blackwell [3]. In characterizing the lateral molecular organization in Technora^®^ from XRD, the researchers carefully noted that the broad lateral apparent crystallite “peak” in Technora^®^ was likely not a single peak, but rather an aggregation of many individual peaks [3]. Ferreira et al. made similar observations from XRD and concluded that this broad peak reflected an “ordered but not crystalline” fiber structure [6]. In addition, analytical modeling of XRD spectra suggested that the three principal monomers that comprise Technora^®^ are randomly sequenced, a conclusion later supported by extensive NMR studies [2,5]. Despite this highly unique chemical structure, research has shown that Technora^®^ and Kevlar^®^ K29 fibers possess similar elastic moduli and tensile strengths [6–7]. These studies also show that Technora^®^ exhibits a narrower distribution in tensile strength, as well as greater creep and fatigue resistance [6–7]. Separate studies of fiber multifunctional properties also showed that Technora^®^ exhibits resistance to moisture and chemical degradation [1,8].

The aforementioned studies have provided critical insights into the structure–property relationships of Technora^®^ fibers. However, our understanding of Technora^®^ would greatly benefit from direct, real-space characterization of the internal structures of these fibers. Real-space measurements could effectively integrate observations of features at different length scales and verify the applicability of analytical structural models used to date. Over the last several years, a “focused ion beam (FIB) notch” technique has been developed and employed to address these gaps in understanding of the internal structures of fibers such as Kevlar^®^ and UHMWPE [9–13]. Here we extend this technique to Technora^®^ by notching individual fibers (Figure 1b), gently opening them along shear planes to expose internal surfaces, and then scanning across those surfaces using an atomic force microscope (AFM) (Figure 1c). AFM scans yield internal nano- and microscale topography, stiffness, and transverse elastic modulus (*E*_T_) maps of internal structural features and local mechanical responses in real space. (See the Experimental section for additional details.) As Technora^®^ has often been compared to Kevlar^®^ K29 fibers, we focus further on comparing the internal structures of these two fiber classes and relate our observations to extensive mechanical testing reported in the literature. Our findings shed new light on the unique interior morphologies of Technora^®^ terpolymer fibers and, in turn, highlight important considerations for the development of future classes of high-performance fibers.

## Results

### AFM characterizations of Technora^®^

**Figure 2:**
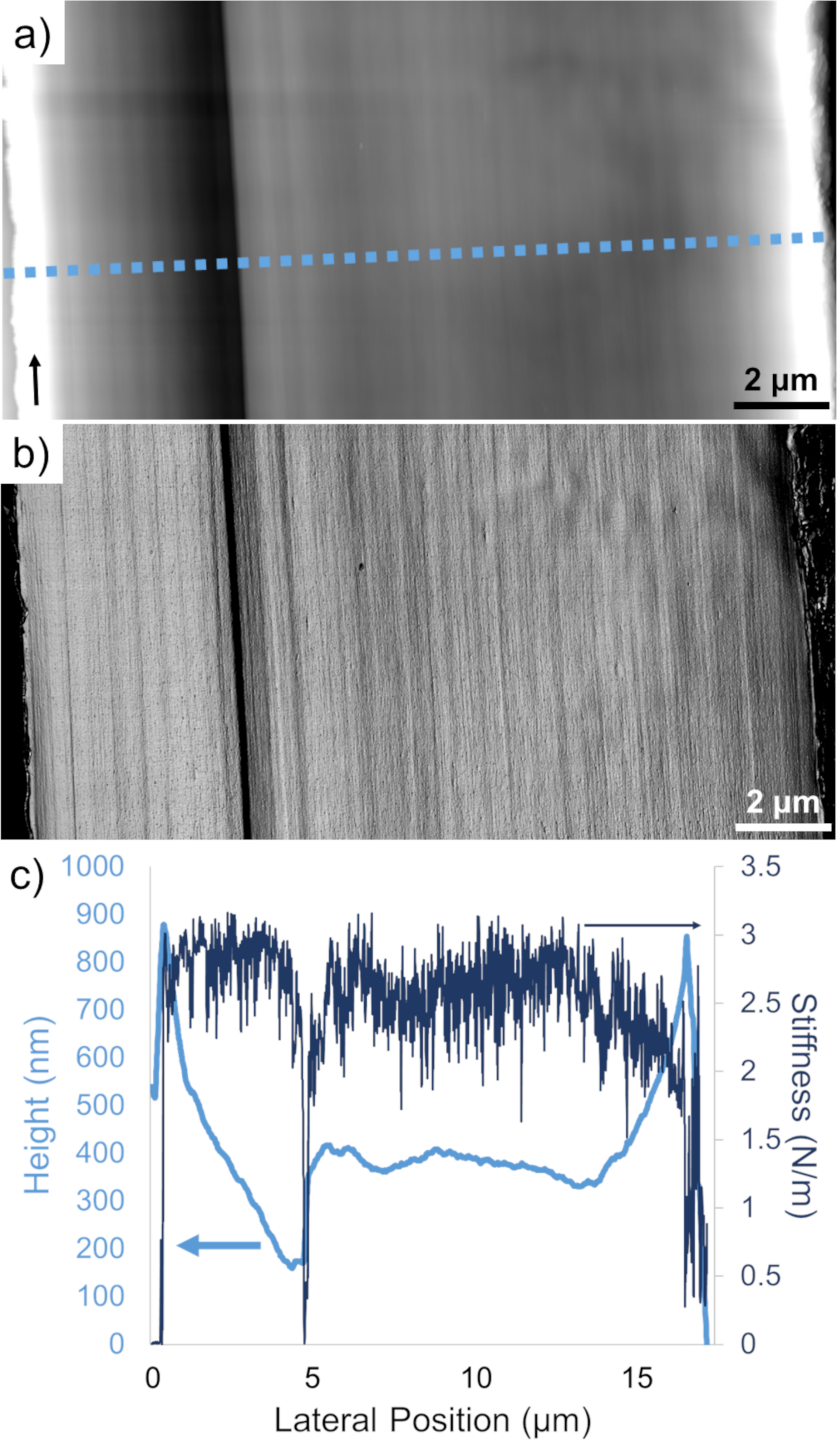
Fiber-wide Technora^®^ AFM maps. (a) Topography map across a full fiber cross section. The arrow (bottom left) denotes the longitudinal direction (i.e., fiber axis direction), while the lateral dashed line denotes the line profile in (c). (b) Stiffness map across a full fiber cross section. (c) Representative lateral topography and transverse stiffness line scans (bright and dark lines, respectively).

AFM maps enable direct characterization of both fiber nano- and microstructures in real space. Large-scale maps span across the fiber diameter to highlight prominent microstructural features. For Technora^®^, both the topography and stiffness maps (Figure 2) revealed a consistent microstructure across the fiber diameter at this length scale. Complementing topography and stiffness maps, lateral line profiles (e.g., dashed line, Figure 2a) quantified both topography and stiffness variations across the diameter. Topography and stiffness (Figure 2c) were remarkably uniform across the diameter as well.

Only two notable features deviate from our consistent topography and stiffness measurements: (i) slopes from the outer edges inward and (ii) a sudden jump at a lateral position of ca. 5 μm. It should be noted that the slopes are very shallow in real space, with height variations of ca. 700 nm or less spanning several micrometers along the diameter. The sudden jump correlates precisely with the lone compliant (dark) longitudinal band in the stiffness map. Based on comparisons with other fibers, we expect that these features resulted from the way this particular fiber split open after FIB notching. Likewise, the lone drop in stiffness makes sense, as the AFM probe experiences a local reduction in tip–substrate contact area. However, similar topography and stiffness jumps forming a compliant band were not observed elsewhere in the fiber.

Topography and stiffness maps also reveal constituents throughout the microstructure that preferentially align with the main fiber axis, as parallel longitudinal lines on topography and stiffness maps can be traced from the top to the bottom of each map. No apparent skin–core differentiation in the microstructure (e.g., as in Kevlar^®^) is observed [11,13–16]. The lack of evidence for a skin–core structure in Technora^®^ was referenced in an earlier study by Derombise et al. [17], but to our knowledge, this is the first time this has been directly shown through real-space mapping.

**Figure 3:**
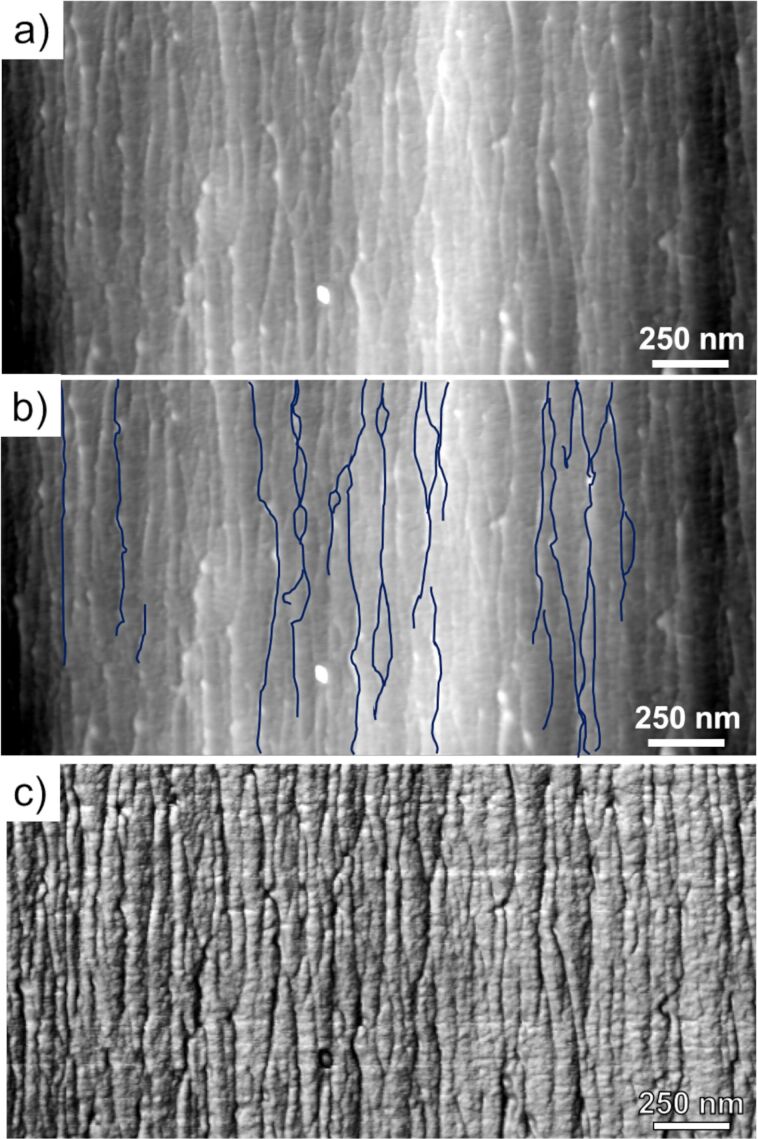
AFM maps of the internal nanostructure within a Technora^®^ fiber. (a) Topography map, 2.5 × 1.25 μm. (b) Copy of the topography map with selected fibrils marked in blue highlighting the branched structure of fibrils aligned with the fiber axis. (c) Stiffness map of the same domain. Scale ranges for each scan are approximately (a, b) 0–12 nm and (c) 0.5–2.5 N/m dark to light.

Complementing these full-fiber scans, high-resolution topography and stiffness maps on smaller fiber subdomains were also obtained to study the nanostructure of Technora^®^. From detailed topography maps, we found that the well-aligned surface features observed on the full-diameter map were fibrillar in structure (Figure 3a). The surface consisted of a branched network of fibrils closely aligned to the fiber axis, as highlighted in Figure 3b. The widths of individual fibrils varied substantially (20–45 nm) along their length. Unlike in other polymer fibers we have explored to date, in Technora^®^, these features regularly merge and separate from one another, intersecting at nodes and splitting off into fibrils with distinct dimensions [9–13]. Likewise, some fibrils appear to protrude into and out from the primary surface exposed by FIB notching, suggesting that this branched network exists in three dimensions within the fiber.

**Figure 4:**
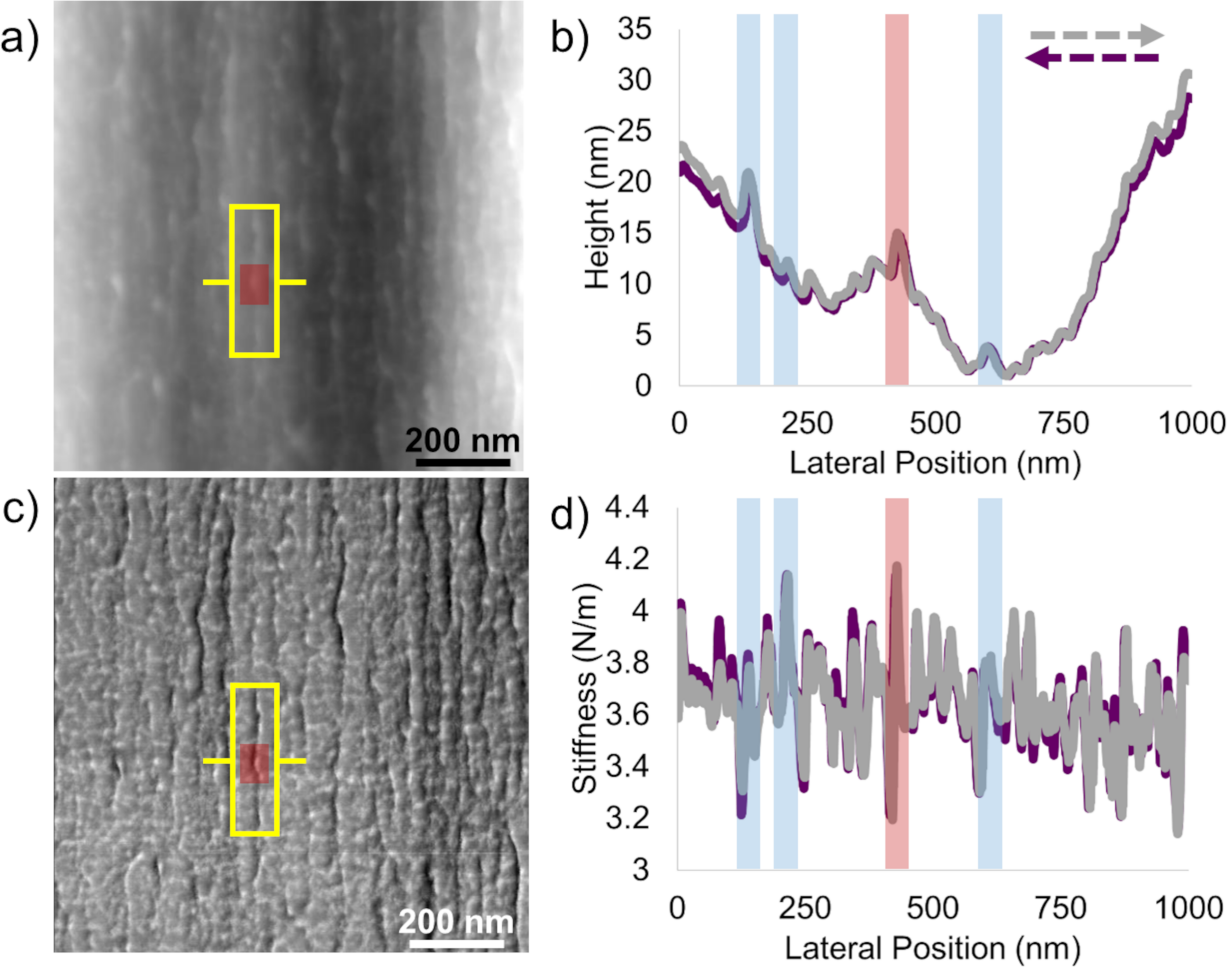
Quantitative AFM maps of internal Technora^®^ fiber nanostructures. (a) Topography map of 1 μm × 1 μm region within a Technora^®^ fiber. (b) Left-to-right trace and retrace line scans through the red box in (a). The peak shaded in red corresponds to the prominent fibril highlighted in (a), while peaks shaded blue highlight repeated observations in other peaks. (c) Stiffness map over the same 1 μm × 1 μm region. (d) Stiffness trace and retrace profiles corresponding to the topography profiles in (b).

Combining these topographical observations with stiffness maps (Figure 3c), a few important features emerged. Along the fiber axis, we observed longitudinal bands of high topography and, nearby, longitudinal bands of low stiffness. Stiffness maps also revealed bands of high stiffness spanning hundreds of nanometers laterally (i.e., left-to-right in each map), along with slightly elevated topographical ribbons. To assess the repeatability of these measurements, we analyzed several nanoscale maps in other representative regions of Technora^®^ fibers and compared trace and retrace profiles (Figure 4). One example is highlighted in the red box (Figure 4a,c) that corresponds to the red shaded part of the profile measurements (Figure 4b,d). Additional examples of these observations in the profiles are highlighted in blue but not imposed on the AFM maps for clarity. In each highlighted region, there was clearly a correlation between topography and stiffness, but the precise nature of this correlation required higher resolution analysis of line profiles.

**Figure 5:**
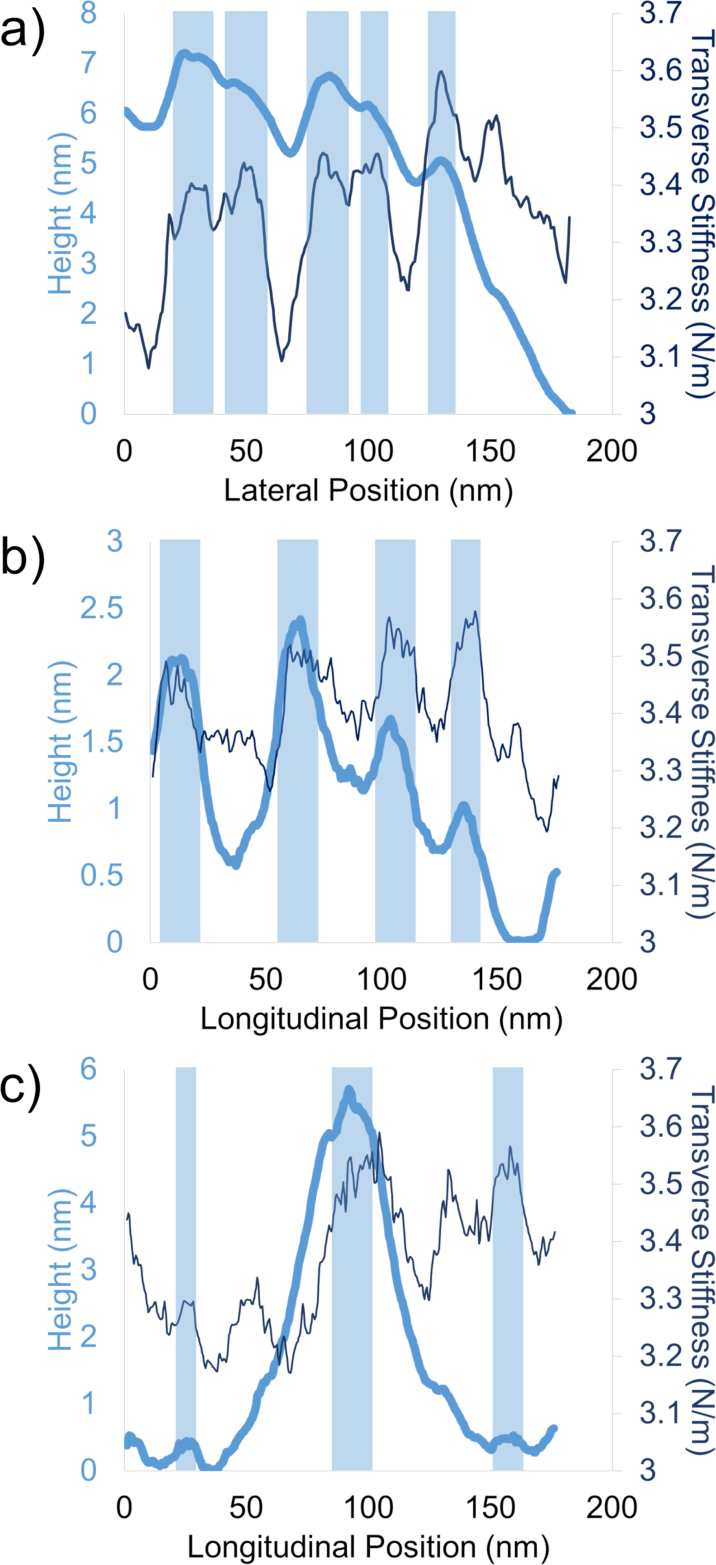
Nanoscale topography–stiffness profile correlations. (a) Representative lateral line scan. (b) Representative longitudinal line scan. (c) Longitudinal line scan over a node. Thick light lines correspond to topography (height), while thin dark lines denote transverse stiffness. In all cases, shaded boxes highlight correlations between locally elevated topography domains and increases in transverse stiffness.

From lateral profiles such as Figure 5a, we observed that domains with high topography directly corresponded to high stiffness. Those lateral profiles indicate that low-stiffness longitudinal bands in the red boxes in Figure 4 do not coincide precisely with the fibrils; instead, they are slightly offset. The manner in which these compliant regions are offset from the fibrils appears to be random. They are not consistently to the left or right of the fibrils, and the offset persists regardless of the AFM probe scanning direction (i.e., trace or retrace, Figure 4). In longitudinal line scans (Figure 5b), we found the same topography–stiffness correlation. Topographically high regions were also stiff. This profile confirms what appear as “ladder rungs” in the blue boxes, supporting the presence of a branched network. These off-axis structures appear fainter than the axial fibrils because of their smaller relative changes in stiffness and height. Finally, to probe the nature of the nodes between branches in the fibril network, we obtained profiles across nodes as well (Figure 5c). Clearly, nodes are substantially taller (ca. 5 nm) than other features along both the longitudinal and lateral directions (1–2 nm), reflecting the fact that discrete fibrils merge. The locally elevated stiffness also reaches the same value as individual fibrils and “ladder rungs”. As the stiffness of the material indirectly reflects the underlying structure, these comparable stiffness values suggest that the material organization in nodes matches that within individual fibrils. Together, these findings provide quantitative details about the branched network of fibrils that defines the nanostructure of Technora^®^.

### Technora^®^ vs Kevlar^®^ K29

**Figure 6:**
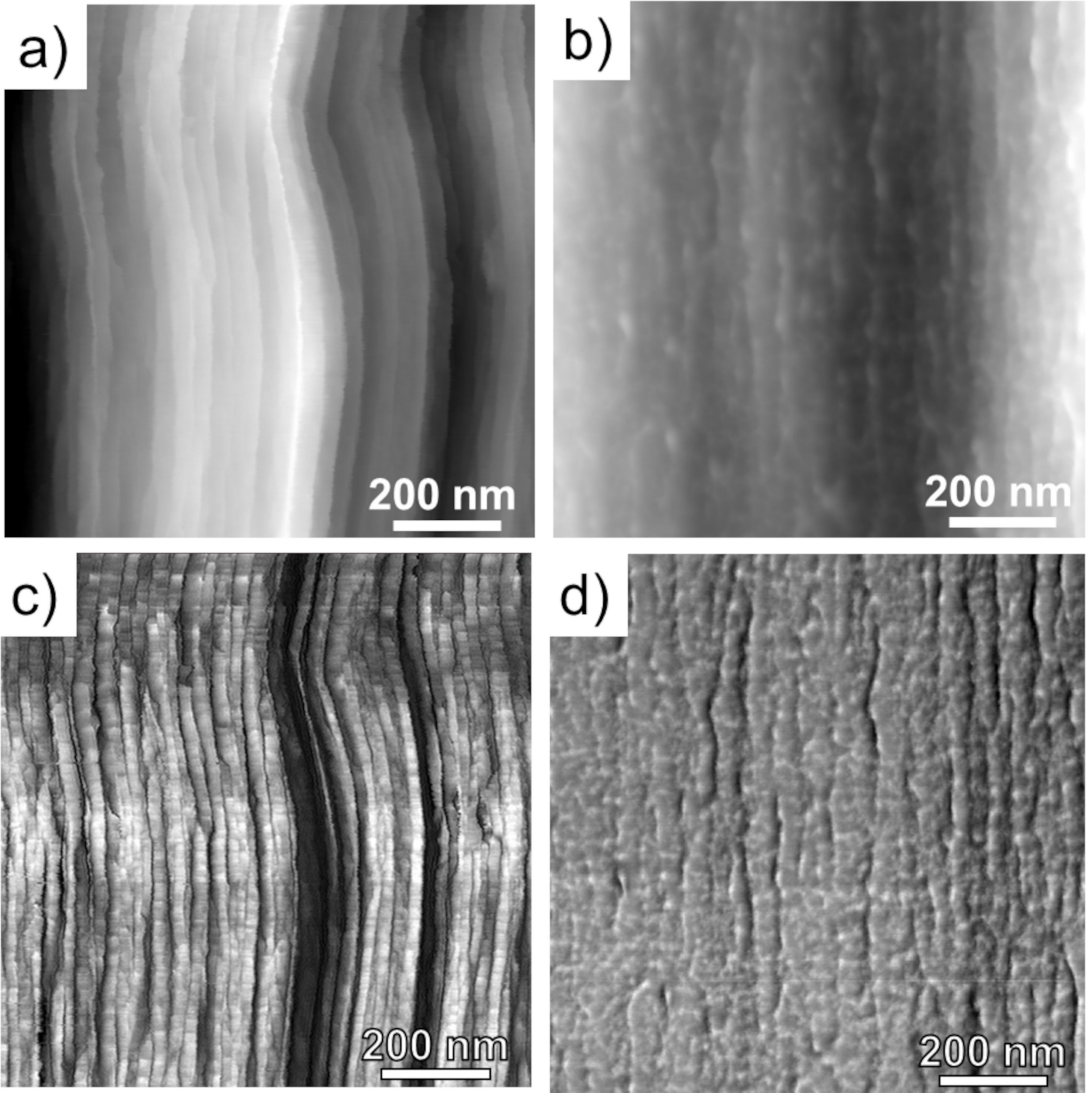
Internal AFM maps of Kevlar^®^ K29 vs Technora^®^. (a, b) Topography maps of Kevlar^®^ and Technora^®^, respectively. (c, d) Corresponding transverse stiffness maps of Kevlar^®^ and Technora^®^, respectively. Scale ranges for each scan are approximately (a) 0–200 nm, (b) 0–30 nm, (c) 0–0.2 N/m, and (d) 2.6–4.4 N/m dark to light. Figure 6c was adapted from [12] (© 2018 E. Sandoz-Rosado et al., distributed under the terms of the Creative Commons Attribution 4.0 International License, https://creativecommons.org/licenses/by/4.0/).

To further highlight the unique internal nanostructure of Technora^®^, we compare topography and stiffness maps of Technora^®^ to the more widely studied Kevlar^®^ K29 [9,11–13]. In particular, we focus on representative 1 μm × 1 μm subdomains inside fibers from each class. In topography maps (Figure 6a), Kevlar^®^ possesses well-packed, distinguishable, 20–25 nm wide fibrils that align closely with the fiber direction. Sharp lateral variations in topography in the lateral direction are observed, akin to vertical steps from left to right, which indicate the interfaces between adjacent fibrils. Fibrils also oscillate with respect to the fiber axis, both laterally and “vertically” (i.e., into and out of the surface plane), reflecting the well-known pleated sheet microstructure within Kevlar^®^ [14]. In contrast to this morphology, Technora^®^ (Figure 6b) shows thin, separated fibrils that are not arranged in close concert with one another. Various fibrils exhibit small oscillations with respect to the fiber axis and protrude into and out of the underlying surface, suggesting a random misorientation of constituents compared to the densely packed and well-oriented sets of fibrils in Kevlar^®^. The aforementioned “branched network” of fibrils is also apparent in Technora^®^ but not in Kevlar^®^.

Comparing corresponding stiffness maps over each domain provides additional insights into these fiber nanostructures. Fibrils in Kevlar^®^ stiffness maps (Figure 6c) were clear with compliant longitudinal bands at the interfaces between adjacent fibrils. In addition, gradations in transverse stiffness were observed in the longitudinal direction, coinciding with adjacent pleat components in the microstructure (ca. every 250 nm along the fiber axis) [11,13–14]. However, Technora^®^ stiffness maps over the same area (Figure 6d) exhibited “mosaic” topography and stiffness maps.

**Figure 7:**
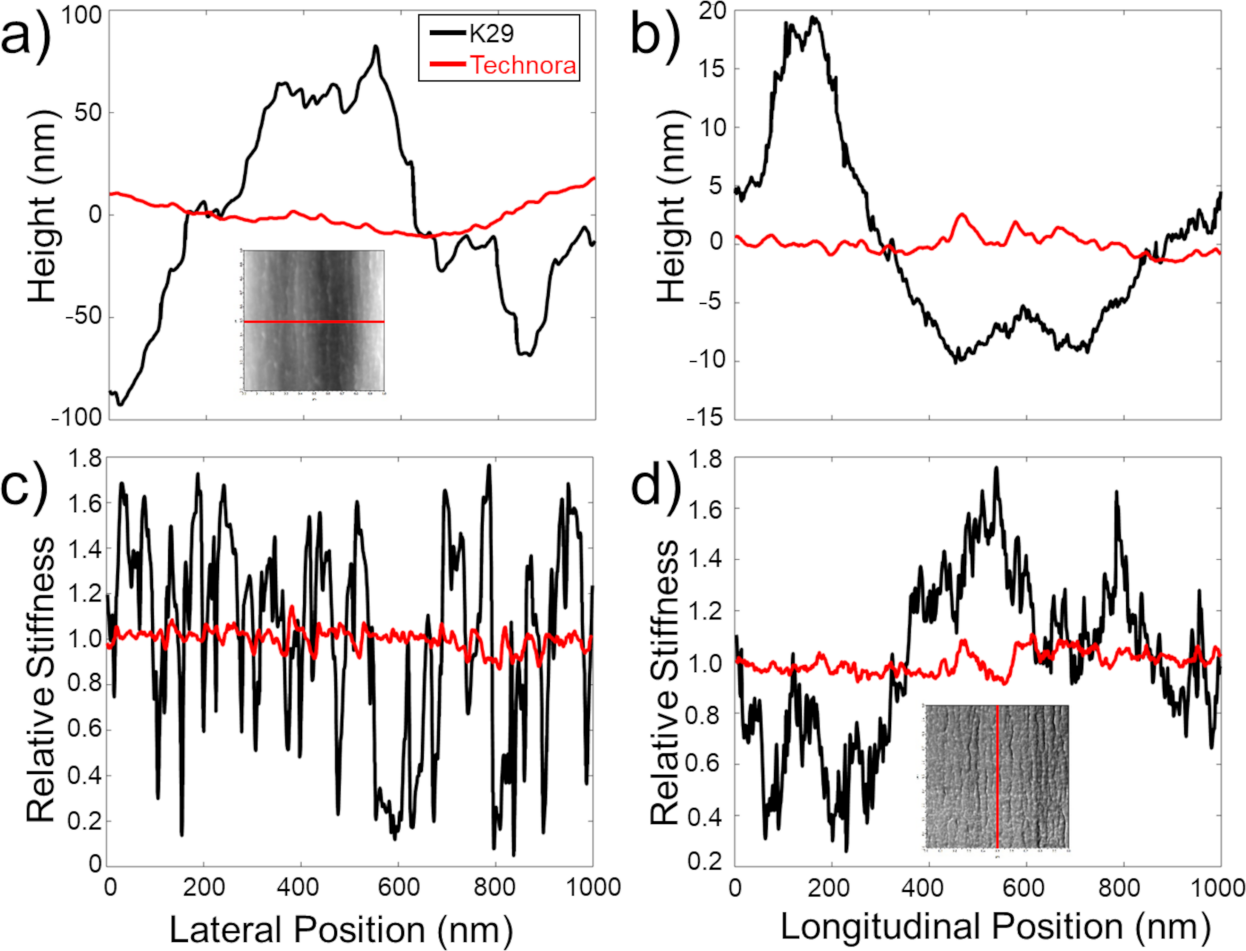
Line profiles across Kevlar^®^ and Technora^®^ subdomains. (a) Overlaid lateral profiles in Kevlar^®^ K29 and Technora^®^ across topographical maps. The inset highlights the line in the Technora^®^ topography map corresponding to the profile shown. (b) Representative longitudinal topographical line scans within the same maps. (c) Lateral line scans of transverse stiffness, corresponding to the topographical scans in (a). (d) Longitudinal transverse stiffness scans corresponding to lines in (b). The inset highlights the vertical line in the Technora^®^ stiffness map.

To further characterize and compare these nanostructural features in Kevlar^®^ and Technora^®^, we quantitatively analyzed both lateral and longitudinal line scans within each of these maps. Lateral topography line scans in Kevlar^®^ (Figure 7a) showed sharp jumps of up to tens of nanometers near fibril interfaces, likely reflecting interfaces between “stacks” of fibrils [11,18]. In contrast, Technora^®^ lateral topography scans over the corresponding domains were much flatter, with roughly an order of magnitude smaller fluctuations in height. Similar results were found in longitudinal topography profiles (Figure 7b), that is, Technora^®^ structural variations along the fiber axis were substantially lower than those in Kevlar^®^. Whereas the out-of-plane pleated sheet microstructure in Kevlar^®^ introduces topological variations of tens of nanometers along the fiber direction, Technora^®^ profiles were smooth and lacked prominent microstructural motifs.

Closely related observations can be made from stiffness maps of the two fibers. From lateral profiles (Figure 7c), Kevlar^®^ transverse stiffness at fibril interfaces dropped down to ca. 90% of the mean stiffness value. While longitudinal Kevlar^®^ transverse stiffness variations (Figure 7c) were not as significant as lateral variations, they still varied up to ±60% of the mean and correlated well with components of the “pleated sheet” internal structure [11,13–14]. In contrast, maximum variations in Technora^®^ transverse stiffness were less than ±15% of the mean both laterally and longitudinally and showed no recurring patterns in stiffness variations.

### Transverse elastic modulus quantifications

We present these last results as relative stiffness variations because multiple AFM tips needed to be used to survey these multiple subdomains. Monitoring tip wear is cumbersome when targeting a broad survey of different regions of multiple fibers, so the results above only reflect a semi-quantitative, relative comparison. To complement these qualitative comparisons, we also quantitatively compared the transverse elastic modulus (*E*_T_) obtained on suitable subdomains within Technora^®^ and Kevlar^®^ K29 fibers.

**Figure 8:**
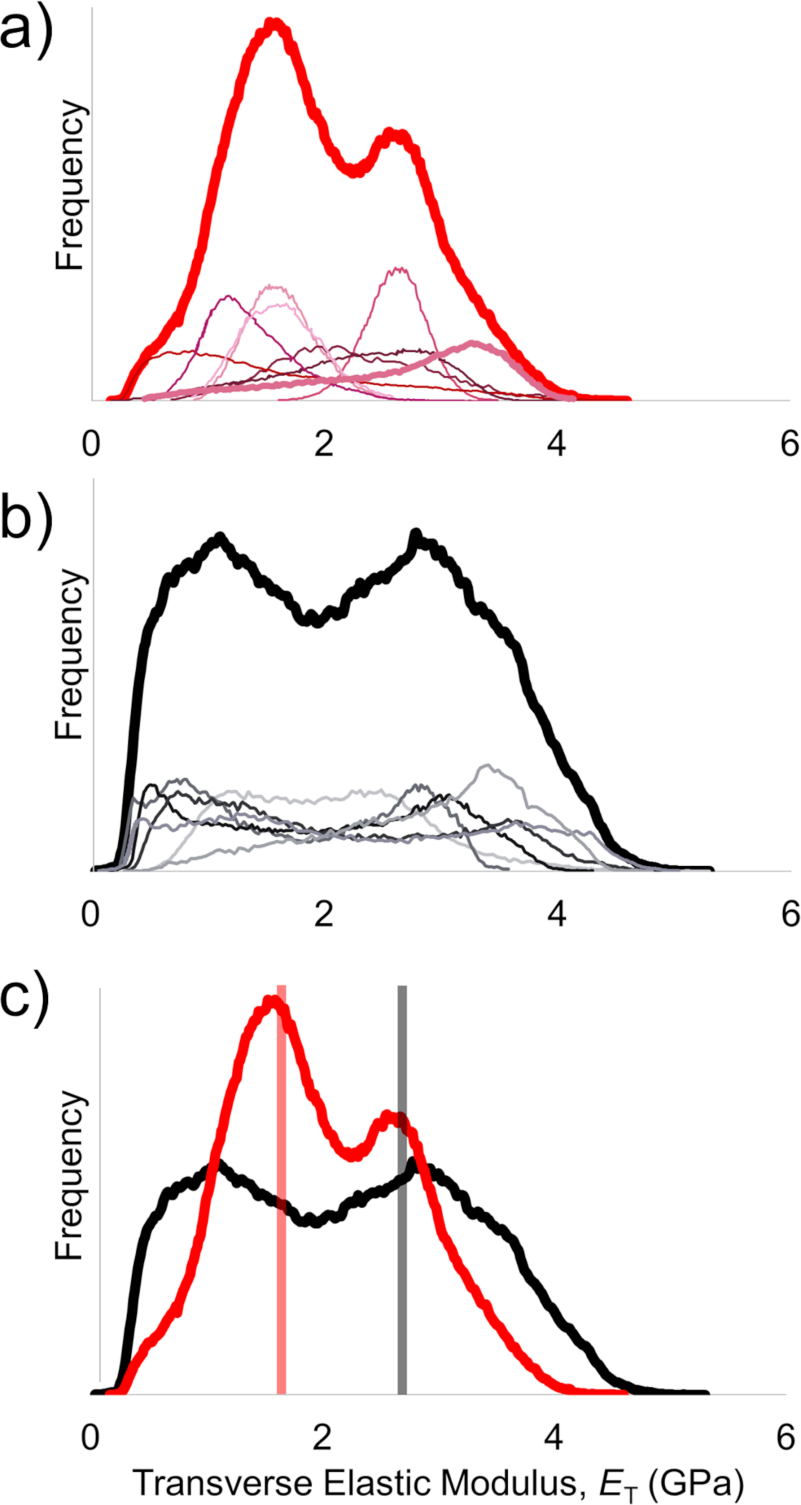
Transverse elastic modulus distributions from multifrequency AFM scans. (a) Frequency distributions from individual Technora^®^ scans, as well as total distribution across all scans (thick red line). (b) Corresponding individual distributions and total distribution from K29. (c) Comparison between total frequency distributions. Solid vertical lines denote mean bulk values reported from published microscale experiments [8,19].

Figure 8 highlights aggregate frequency distributions in transverse elastic modulus (bold top lines) and individual distributions (lower lines) obtained from multiple subdomains of Technora^®^ and Kevlar^®^ K29 fibers. In Technora^®^, individual distributions (i.e., distributions within any one region) were narrow with clear peaks (Figure 8a). While several scans showed frequent readings near 2.6 GPa, *E*_T_ centered around 1.5–1.6 GPa in most scans. Taken together, this led to a total distribution with a mean of 2.00 GPa and a mode (i.e., highest peak) of 1.53 GPa. In contrast, K29 individual distributions (Figure 8b) appeared as plateaus ranging from 0.5 to 3.5 GPa. When present, shallow peaks in K29 arose near these upper and lower limits, resulting in a plateau in the overall frequency distribution. The mean for K29 was 2.18 GPa. The mode of this total K29 distribution was 2.79 GPa, though a second peak near 1.1 GPa was virtually as likely to occur based on this distribution.

Figure 8c isolates each total distribution to facilitate direct comparisons between them. In addition, vertical shaded boxes in Figure 8c indicate the mean values from full-fiber transverse compression experiments reported in the literature for each fiber class (2.68 GPa for Kevlar^®^, 1.64 GPa for Technora^®^) [8,19]. These average values coincide very closely with the modes of each distribution from our nanoscale AFM measurements.

## Discussion

In this study we have, for the first time, isolated and directly characterized the internal structures of Technora^®^ fibers using AFM. Here we elaborate and contextualize several aspects related to our findings. First, we describe how this real-space characterization provides new information about the Technora^®^ fiber nanostructure. We then focus on fiber microstructures, highlighting how this technique expands upon conclusions from previous studies. Lastly, we comment on how characterizing the structural composition in real space relates to Technora^®^ performance parameters. In particular, through comparisons with the Kevlar^®^ K29 structure, we discuss structure–property relationships that explain the mechanical properties of these two fibers. We also analyze several critical multifunctional performance parameters highlighted by the developers of Technora^®^ and explore how they relate to the fiber structures. Together, we believe these analyses help elucidate structure–property relationships within high-performance polymer fibers.

### Technora^®^ nanostructure

**Figure 9:**
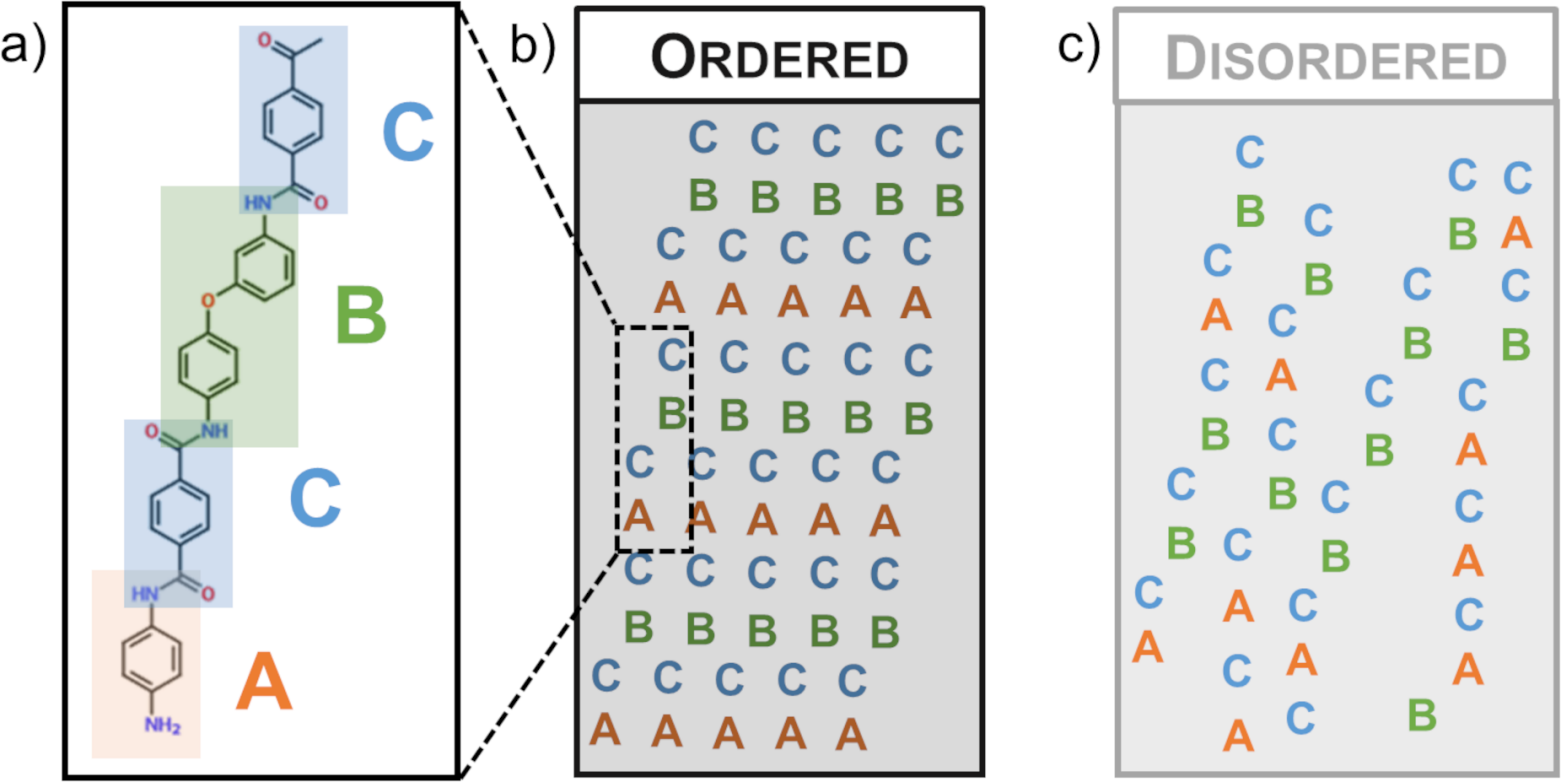
Schematic of possible molecular sequencing in Technora^®^ fibers. (a) With random sequences of molecular subunits (e.g., ACBC, top left), adjacent extended chain molecules can form ordered or disordered subdomains, per theoretical schematics highlighted in (b) and (c). Fibrils directly observed in this study suggest that even some disordered subdomains can have sufficiently strong interactions to form fibrils.

In terms of the Technora^®^ nanostructure, our measurements directly reveal two key motifs: (i) the presence of nanoscale fibrils that are well aligned with respect to the fiber axis and form a branched network and (ii) nanoscale domains with high local transverse stiffness. Through WAXD studies, Ferreira et al. deduced that Technora^®^ fibers are “ordered but not crystalline” [6]. This conclusion aligned well with corresponding WAXD and NMR studies, which indicated that the molecules within Technora^®^ include random monomer sequences [2–3,5]. Adjacent extended-chain molecules would not be able to form crystallites, as random sequences of molecules including the DPE monomer (which locally changes chain orientation) would prevent neighboring molecules from forming cohesive extended crystals. This would lead to a mixture of ordered and disordered subdomains with varying alignment, as schematized in Figure 9. Nevertheless, Ozawa hypothesized that well-aligned, extended chain molecules consisting of 50% PPTA concentration could still effectively transfer applied loads between adjacent chains [1].

These previous characterizations of the molecular chemistry of Technora^®^ appear to explain the branched network of nanoscale fibrils we observe. Despite the randomly sequenced terpolymer chemistry, nanoscale fibrils still clearly form in Technora^®^. The branched network likely results from local variations in the strength of intermolecular interactions. For example, misalignment between adjacent molecules (e.g., due to molecular “bends” within DPE molecules) or weak intermolecular interactions (e.g., due to distributions in monomers capable of intermolecular hydrogen bonding) may make it energetically favorable for sets of molecules to branch apart at different junctures rather than to remain together within a fibril. These unique molecular structures cannot lead to a truly crystalline fiber, but evidently, they are still strong and concentrated enough to form the nanoscale fibrils we repeatedly observe throughout Technora^®^ fibers.

At the same time, a random monomer sequence should result in a heterogeneous map of transverse stiffness within the material. Here the stiffness maps of Technora^®^ sharply contrast with those of Kevlar^®^. In Kevlar^®^, the primary observable nanostructural features are sets of adjacent fibrils with well-defined, compliant interfaces (Figure 6). In Technora^®^, both lateral and longitudinal line scans (Figure 5a,b) show that elevated fibrils exhibit high transverse stiffness. This high stiffness suggests that the material within Technora^®^ fibrils is well-ordered, that is, lacking compliant nanoscale subdomains that may arise from voids, mismatched molecules, or other defects. Furthermore, line scans across nodes (Figure 5c) show virtually the same stiffness as neighboring fibrils, indicating that these nanoscale features can merge and separate without affecting transverse mechanical responses. Overall, our nanoscale characterizations of AFM topography and stiffness maps corroborate earlier findings that suggested Technora^®^ possesses (i) repeated longitudinal features along the crystalline axis and (ii) a broad distribution of apparent lateral crystallite sizes, implying an ordered but non-crystalline structure [3,6]. Moreover, we directly uncover fibril constituents within Technora^®^ fibers and relate their nanoscale structures to previous studies of the fibers’ fundamental chemistry.

### Technora^®^ microstructure

We can also use our real-space AFM scans to highlight two observations regarding the microstructure of Technora^®^ fibers. First, WAXD studies previously suggested that Technora^®^ does not possess the same degree of “three-dimensional order” as Kevlar^®^ [3]. Our line scans over representative 1 μm × 1 μm regions of Technora^®^ and Kevlar^®^ fibers (Figure 7) support this analysis. Discrete fibrils can be clearly traced throughout the core microstructure of Kevlar^®^. At fibril interfaces, we see sudden topographical jumps up to tens of nanometers. This indicates that, when the fiber is separated after FIB notching, the crack that forms the exposed surface follows these interfaces rather than fracturing crystallites within the fibrils. In contrast, the exposed plane within Technora^®^ is significantly flatter and less well-defined, as evidenced by surface fibrils penetrating into and out of the exposed surface. Without forming crystallites in the lateral direction, there appears to be a lack of consistent lateral packing in Technora^®^; thus, the fiber cannot break along well-defined planes. This explains how the complex nanostructure translates up to the microstructure, leading to more disorder in 3D space in Technora^®^ compared to Kevlar^®^.

The other unique microstructural aspect is the lack of a fiber skin in our real-space AFM scans of Technora^®^. In Kevlar^®^, the skin is a prominent feature up to ca. 1 μm thick and readily observable with AFM and other techniques [11,13,16,20–21]. It is expected to arise during Kevlar^®^ fiber processing due to different cooling rates near the surface of the fiber and inside the fiber; consequently, Kevlar^®^ fibers that undergo post-processing heat treatments possess thinner skins [11,13,15]. In previous studies, we corroborated this trend for different classes of Kevlar^®^ fibers using real-space AFM scans, as skin regions contained distinct topographical features and lower stiffness than the Kevlar^®^ fiber core [11,13]. For Technora^®^, however, no such features were ever observed. There are shallow topographical gradations near the outer edges of the fiber (Figure 2c), but these are distinct from the sharp changes at the skin–core interface we observed in Kevlar^®^. Moreover, there are no clear stiffness variations near the periphery compared to the fiber center, suggesting no change in the underlying material structure across the fiber diameter. We expect that this lack of a skin–core structure is yet another manifestation of the non-crystalline structure within Technora^®^, resulting in an ostensibly homogeneous microstructure throughout the fiber.

### Transverse elastic modulus quantifications

We begin our discussion of the transverse elastic modulus measurements by focusing on the distributions in *E*_T_ values (i) within each fiber type and (ii) between the two fiber classes. Both means and modes are important statistical averages to consider in the context of these experiments. The mean provides an overall average mechanical response, but it is more sensitive to impacts of individual scans than the mode, which tells us the *E*_T_ value measured most frequently in different areas. For example, in the overall *E*_T_ distribution in Technora^®^ (Figure 8a), the highest peak (i.e., the mode) is centered at 1.53 GPa, while secondary peaks and shoulders in the distribution arise at other values, for example, at ca. 2.6 GPa and, less prominently, around 0.5 GPa. Yet for Technora^®^, it is interesting to note that multiple individual peaks center around the mode of 1.53 GPa. While the Kevlar^®^ K29 mean *E*_T_ is only 9% higher than that of Technora^®^, the shapes of the individual and total distributions resembled plateaus with shallow peaks. Technically, the mode of the K29 measurements is 2.79 GPa, but another shallow peak in the plateau centered appears at 1.1 GPa.

In contrast to the modulus maps in Technora^®^, the distinctions between the local *E*_T_ measurements within fibrils and their interfaces are very clear in Kevlar^®^. Image thresholding of K29 modulus maps indicates that the most compliant regions are near interfaces, which can also be readily observed from lateral line scans (Figure 7c). However, these interfaces are not uniformly compliant, that is, different interfaces exhibit unique *E*_T_ values. Each one is consistent along the fiber axis (i.e., the stiffness along any particular interface is uniform), but they are not consistent with one another. This compliance likely stems from a local reduction in contact area, as sudden changes in height up to tens of nanometers can occur at these interfaces (Figure 7a). Nevertheless, these are “real” *E*_T_ values; in other words, these local gaps in the fiber would affect *E*_T_ of the full fiber. Individual Kevlar^®^ fibrils typically exhibit their highest *E*_T_ values toward their centers; however, the modulus values among fibrils vary. The apparently independent values of fibrils and their interfaces can explain the overall plateau-like distribution of *E*_T_ in Kevlar^®^. In addition, the fundamental chemistry of Kevlar^®^ (Figure 1) leads to a well-ordered molecular structure (i.e., ‘ACACAC…’ in Figure 9), which should enhance load transfer at this scale compared to Technora^®^. Together, the more uniform molecular sequencing and the resulting microstructure in Kevlar^®^ appear to explain why K29 has higher mean and mode *E*_T_ values than Technora^®^ in our measurements.

Finally, it is important to compare our nanoscale AFM measurements of transverse moduli with full-fiber transverse compression experiments conducted in other studies. We find that the modes from our AFM-based measurements correspond closely with the full-fiber *E*_T_ values from two different studies [8,19]. For Technora^®^, remarkably similar quantitative values (AFM: 1.53 GPa; mean full-fiber: 1.64 GPa) may reflect how the microstructure throughout the fiber (Figure 2) consistently reflects the nanostructure (Figure 4). Thus, we would expect the *E*_T_ measurements to be similar across length scales.

The analysis for Kevlar^®^, however, is more complicated. Our nanoscale *E*_T_ measurements indicate stiffer average mechanical responses in Kevlar^®^ K29 than in Technora^®^, in agreement with the aforementioned full-fiber experiments [8,19]. However, we must acknowledge the plateau-like nature of the distribution in our measurements, with a second peak near 1.1 GPa. Moreover, at the full-fiber level, the skin–core structure in Kevlar^®^ must be considered. Previous studies have suggested that Kevlar^®^ skins are more compliant than the cores [11,13,20–21]. We have implicitly focused on Kevlar^®^ cores, but when full Kevlar^®^ fibers are subjected to transverse compression, we would expect a mixed mechanical response based on both core and skin stiffness. Thus, our broad distributions in nanoscale Kevlar^®^ values and this clear distinction between nano- and microstructural characterization of Kevlar^®^ make this comparison less conclusive than that of Technora^®^. Nevertheless, the fact that these materials exhibit similar transverse elastic responses at the nanoscale and across the full fiber is noteworthy, and appears to support the validity of quantitative materials characterization at the nanoscale with this technique.

### Technora^®^ vs Kevlar^®^ K29: structure–property connections

#### Tensile properties

The structural distinctions between Technora^®^ and Kevlar^®^ K29 have fascinating implications for the tensile mechanical properties of these fiber classes. Studies have shown that their mean single-fiber tensile properties, for example, tensile strength and elastic modulus, are quite similar [6–7]. In addition, Technora^®^ single-fiber tensile strengths are more consistent than those of Kevlar^®^ K29 [6–7]. The similar mean tensile properties appear to support the hypothesis put forth by Ozawa, that is, a well-defined crystal structure is not required for a high-performance polymer fiber, provided that fixing extended-chain molecules and aligning them with the fiber axis can be achieved through other means [1]. Indeed, the tensile strengths of high-performance paracrystalline fibers are nearly an order of magnitude lower than those of their constituent molecules and crystallites, so other structural factors play a considerable role in limiting fiber properties [22]. To this end, the more consistent strength values of Technora^®^ appear to stem from two sources. First, as noted by Morgan et al., a paracrystalline fiber such as Kevlar^®^ will have preferential fracture planes across its core, in agreement with AFM profiles shown here (Figure 7c) that clearly demarcate a fracture path between fibrils [15]. When a crack extends into the fiber core, the failure will be brittle and thus more prone to variation. In contrast, Technora^®^ has no such preferential planes, which may explain its more consistent strength values [6–7]. Indeed, we observe stray fibrils protruding in and out of the FIB-notched surfaces of Technora^®^ and no consistent fracture planes (Figure 7c), which would necessitate fibrils breaking or separating in a manner inconsistent with brittle failure.

Second, the presence or absence of skin–core differentiation likely plays a critical role in dictating the distributions of the tensile strengths of these fibers as well. Şahin et al. suggest that the skin–core interface dominates the ultimate tensile strength of Kevlar^®^, as evidenced by a “sword-in-sheath” pullout of the core from the skin during tensile tests [23]. Kevlar^®^ fibers with larger skins typically exhibit higher tensile strength as well, suggesting that the load-bearing capabilities of these fibers are significantly diminished once a crack permeates the skin [11,13,16]. This interface appears to be another mechanism that leads to a somewhat brittle failure in Kevlar^®^, resulting in a distribution in single-fiber tensile strengths. In contrast, the lack of skin–core differentiation in Technora^®^, evidenced here via real-space scans, should also lead to more consistent strength values.

#### Additional mechanical properties

Significant connections can also be made between the structures of Technora^®^ and Kevlar^®^ fibers and other mechanical properties, such as creep and fatigue. Ferreira et al. demonstrated that, while neither Technora^®^ nor Kevlar^®^ fibers exhibit significant amounts of creep deformation, Technora^®^ fibers creep slightly less [6]. They hypothesized that this distribution stems from more local links between adjacent molecular chains in Technora^®^ than between crystallites in Kevlar^®^ [6]. Extending this molecular-level hypothesis, it is likely that the highly fibrillated nanostructure in Technora^®^ (Figure 3 and Figure 4) has the same effect. Local, randomly distributed load transfers between fibrils could more effectively inhibit creep at this length scale than highly aligned fibrils such as those in Kevlar^®^. Creep behavior could also be affected considerably by the microstructures of the fibers. In Kevlar^®^, the well-defined pleated microstructure would be prone to deform under creep, while the heterogeneous Technora^®^ microstructure would be less likely to deform in a consistent manner throughout the fiber [24].

These structure–property connections appear to correlate with the fatigue behavior of these fibers as well. Ferreira et al. found that Technora^®^ fibers exhibited the same tensile moduli through 80 load cycles, while Kevlar^®^ K29 fiber moduli changed several times under cyclic loading [6]. Most notably, after 20 cycles, a new regime of lower tensile modulus under high loads was observed in Kevlar^®^, which the authors attributed to interfaces between PPTA crystallites breaking irreversibly [6]. Ozawa also found that Technora^®^ was more resistant to fatigue than Kevlar^®^ and attributed this difference to two fundamental molecular mechanisms: the more compliant molecular chain and the “less developed” crystal structures in Technora^®^ [1]. These molecular-level features likely explain the nanoscale features in our AFM maps, as discussed earlier. In turn, it appears that this heterogeneous structure makes Technora^®^ fibers less prone to both creep and fatigue than Kevlar^®^.

#### Multifunctional properties

Beyond establishing connections between fiber structures and mechanical properties of Technora^®^ and Kevlar^®^, it is also important to highlight how structural features relate to multifunctional properties. One such property is resistance to chemical attacks. By measuring the tenacity (i.e., tensile strength) of Kevlar^®^ K29 and Technora^®^ fibers immersed in both basic and acidic solutions, Ozawa found that Technora^®^ fibers are typically an order of magnitude more resistant to chemical attacks than Kevlar^®^ K29 [1]. A small but important subset of ambient “chemical” attacks includes moisture sensitivity. Ozawa observed substantially larger tenacity reductions in Kevlar^®^ than in Technora^®^ as a function of ambient moisture [1]. Minoshima et al. also found that Kevlar^®^ K29 fibers absorbed about 6.6 times more deionized water than Technora^®^ when immersed, which resulted in larger reductions in full-fiber *E*_T_ for Kevlar^®^ [8]. These findings appear to reflect the distinct fiber microstructures. In Kevlar^®^, the well-defined pleated sheet structure allows for preferential planes through which intercalating chemicals can permeate and interact with the material [1,15]. This is particularly true for the interfaces we observe between fibrils, where needle-like voids can coalesce and create ideal planes for chemical infiltration [25]. In contrast, the branched filaments within Technora^®^ fibers present no such consistent internal planes. Indeed, Ozawa hypothesized that Kevlar^®^ has much wider “internal surfaces” than Technora^®^, making the former more sensitive to chemical effects [1]. This hypothesis aligns very well with our line scans (Figure 6), which show Kevlar^®^ exhibiting longer and more clearly defined interfaces than Technora^®^. Thus, through real-space AFM measurements, we have expanded upon previous hypotheses by uncovering how these internal morphological features take shape, helping to explain how structures affect fiber properties.

## Conclusion

Here we present internal structural characterizations of FIB-notched Technora^®^ fibers using multifrequency AFM mapping. We observe a homogeneous microstructure throughout the fiber without any evidence of a fiber skin near the periphery. At the nanoscale, we observe a highly fibrillated structure well aligned with the fiber axis with significant fibril branching, junctions, and weaving in and out of the exposed surface. Combining topography maps with stiffness maps, we find that locally elevated regions exhibit higher stiffness, regardless of scanning direction, orientation with the fiber axis, or the relative heights of different sections (e.g., single fibrils versus nodes between multiple fibrils).

Our nano- and microscale characterizations of Technora^®^ are further aided by extensive comparisons with Kevlar^®^ K29, which possesses similar mechanical properties despite a significantly different internal structure. Comparisons of AFM maps from each fiber class revealed that the Technora^®^ nanostructure includes (i) more fibril branching, (ii) less-defined interfaces, and (iii) more homogeneous stiffness profiles than in Kevlar^®^ K29. These semiquantitive comparisons of stiffness were further supported by direct quantification of transverse elastic modulus (*E*_T_) of both fiber types, which showed more consistent *E*_T_ measurements in Technora^®^ than in Kevlar^®^ K29. Nanoscale *E*_T_ measurements in Technora^®^ also matched exceptionally well with previously reported macroscopic measurements, which would be expected for a homogeneous microstructure. In contrast, our nanoscale measurements did not match as well with macroscopic measurements of Kevlar^®^ K29, perhaps as a result of the skin–core structure present in Kevlar^®^ K29 but absent in Technora^®^.

Taken together, our characterizations uncover the internal structures of these fiber classes, which in turn help explain the mechanical and multifunctional performances of these fiber classes reported in other studies. In the future it would be especially interesting to carry out higher resolution scans within these fibers to characterize atomic structure [26]. Flat Technora^®^ subdomains may be especially amenable to this characterization, which could shed more light on the sequencing of molecular subdomains (Figure 9) discussed here and in previous reports [2–5]. In addition, it would be interesting to apply the FIB notch technique to Kevlar^®^ and Technora^®^ fibers that underwent mechanical conditioning (e.g., fatigue) and/or chemical treatment to complement prior characterizations and improve our understanding of the mechanisms that determine the multifunctional performance of these fibers.

## Experimental

### Materials

Individual Technora^®^ T-240 fibers were extracted from a 65 decitex yarn. Fibers were mounted onto cardstock specimen holders, fixed with adhesive at two points approximately 15 mm apart from one another, and conductively coated with a gold–palladium mixture to mitigate sample drift, for example, due to sample charging effects inside the FIB chamber [9]. Kevlar^®^ K29 fibers also discussed in this report underwent the same preparation techniques.

#### Focused ion beam notching

Conductively coated fibers were cut with a FIB as discussed in detail in an earlier study [9]. In summary, 2–3 μm wide through-cuts were notched into each fiber (Figure 1b), extending halfway up or down the fiber diameter on opposing ends. This creates a shear plane that readily splits the fiber under slight tension, exposing internal surfaces that can be probed with AFM. Notches were obtained using a gallium ion beam inside an FEI NanoV600 dual beam system, with ion beam voltage and current of 16 kV and 3.3 nA, respectively. After FIB notching, specimens were mounted onto magnetic discs with double-sided tape as shown (Figure 1c) for AFM characterization [9].

The internal fiber surfaces produced by FIB notching closely match corresponding surfaces obtained using other techniques, as previous studies have shown for Kevlar^®^ [9,11,14]. While microtomy can also be used to access internal fiber surfaces, marks from the microtome blade often remain visible [9], which is a disadvantage compared to FIB-notched surfaces. However, each FIB notch must be carefully prepared so that it laterally cuts through half of the diameter, or slightly more. Undercutting prevents a shear plane from forming, yielding a highly fibrillated internal surface that cannot be characterized with AFM, as stray fibrils protrude from the surface and move when contacted by the tip. In contrast, overcutting leads to transverse failures near the notches without producing an internal shear plane.

#### Multifrequency atomic force microscopy scanning

The exposed internal surfaces of FIB-notched fibers were scanned using a Cypher AFM with an ARC2 controller (Asylum Research). Olympus AC200TS cantilevers (*k* ≈ 9 N/m (nominal), k = 12–21 N/m (measured)) were used for AFM scans. By simultaneously exciting the AFM cantilever to its first and second resonance (*f*_1_ = 160–190 kHz and *f*_2_ = 900–1150 kHz, Figure 1c) and applying two distinct control conditions (amplitude modulation (AM) and frequency modulation (FM) to the first and second modes, respectively), maps of surface topography, transverse stiffness, and transverse elastic modulus across the fiber were obtained. Typical (setpoint/free air) amplitudes for the first mode were ca. 25/50 nm. Second mode amplitudes (always held constant, in and out of contact) were approximately 1–2 nm. Some images were enhanced by approximately doubling both the setpoint and free air amplitudes while maintaining the amplitude ratio close to 50%. Typical map sizes and resolutions ranged from 500 nm × 500 nm (512 × 512 pixels, ca. 1 nm/pixel) to 20 × 20 μm (2048 × 2048 pixels, ca. 10 nm/pixel), depending on whether the map was intended to characterize fiber nanostructure or microstructure.

To clarify, both transverse stiffness and transverse elastic modulus maps obtained with AFM provide information about the local mechanical response of the material. Transverse stiffness (or “stiffness”), quantified in [N/m], makes no assumptions about tip–substrate interactions such as tip size or shape. It is primarily useful for semi-quantitative mapping to show the relative fluctuations in stiffness within different regions. However, stiffness itself is not a material property, that is, using AFM tips with different shapes and sizes, or worn tips, will yield different stiffness values. Thus, when presenting transverse stiffness values, we focus on the relative variations within different maps.

Maps of transverse elastic modulus (*E*_T_), quantified in [GPa], directly quantify nanoscale material properties of the fiber surface, though they present additional challenges versus stiffness maps that must be addressed. In particular, tip wear has to be carefully monitored to ensure that the tip radius is well characterized in each scan. Here we focused *E*_T_ analysis on small fiber subdomains (typically 500 nm × 500 nm, ca. 1 nm/pixel) to reduce the likelihood of tip wear. In addition, subdomains were initially mapped out with “sacrificial” AFM tips, and *E*_T_ distributions were only then obtained from scans with fresh, well-characterized AFM tips. In quantitative scans, the shape of each AFM tip was taken to be a cylindrical punch, and the tip radius was calibrated by scanning on polystyrene (*E*_T,PS_ = 2.7 GPa, Bruker) as a control substrate before and after each scan on a fiber surface. *E*_T_ quantifications from fiber maps were only kept when mean *E*_T,PS_ values varied by less than 10% between the pre- and post-scans on polystyrene.

To ensure repeatability of measurements and observations, AFM maps and quantitative data were each obtained from at least one region in two separate Technora^®^ and two separate Kevlar^®^ fibers. The *E*_T_ measurements were the most restrictive considering the need for pre- and post-scans to confirm calibration. Additional scans were obtained from other regions within the same fibers, as well as other fibers, and we observed similar features to those reported here. For clarity, our analysis has focused on regions that were most clearly defined in AFM scans.

Line profiles, *E*_T_ distributions, and other quantifications of AFM maps were characterized using the Gwyddion software package [27]. Additional details on multifrequency AFM mapping theory, analysis and applications have been provided in earlier literature reports [10,28–29].
